# Dietary *Arthrospira platensis* improves systemic antioxidant potential and changes plasma lipids without affecting related hepatic metabolic pathways in *post*-weaned piglets

**DOI:** 10.1186/s12917-021-02869-y

**Published:** 2021-04-13

**Authors:** Marta Sofia Morgado dos Santos Madeira, Paula Alexandra Antunes Brás Lopes, Cátia Falcão Martins, José Miguel Pestana Assunção, Cristina Maria Riscado Pereira Mateus Alfaia, Rui Manuel Amaro Pinto, José António Mestre Prates

**Affiliations:** 1 CIISA - Centro de Investigação Interdisciplinar em Sanidade Animal, Faculdade de Medicina Veterinária, Universidade de Lisboa, Pólo Universitário do Alto da Ajuda, Av. da Universidade Técnica, 1300-477 Lisbon, Portugal; 2 LEAF - Linking Landscape, Environment, Agriculture and Food, Instituto Superior de Agronomia, Universidade de Lisboa, Tapada da Ajuda, 1349-017 Lisbon, Portugal; 3 iMed.UL, Faculdade de Farmácia, Universidade de Lisboa, Avenida Professor Gama Pinto, 1649-003 Lisbon, Portugal

**Keywords:** *Arthrospira platensis*, Enzymes, Antioxidant potential, Hepatic lipid metabolism, Piglets

## Abstract

**Background:**

The ability of a high level of dietary *Arthrospira platensis*, individually or in combination with two exogenous carbohydrate-degrading enzymes (lysozyme and Rovabio^®^), to improve systemic antioxidant potential and hepatic lipid metabolism was tested in piglets. Forty male *post*-weaned piglets, sons of Large White × Landrace sows crossed with Pietrain boars, were allocated into 4 groups (*n* = 10) and fed during 28 days one of the following diets: 1) a control basal diet (cereal and soybean meal); 2) a basal diet with 10% of *A. platensis* (AP); 3) the AP diet supplemented with 0.005% of Rovabio^®^ (AP + R); 4) the AP diet supplemented with 0.01% of lysozyme (AP + L).

**Results:**

*Arthrospira platensis* decreased BW gain of piglets, regardless the addition of feed enzymes. The majority of plasma metabolites were affected by diets. *A. platensis* increased total lipids, total cholesterol and LDL-cholesterol, without changing hepatic fatty acid content or modulating, in an expressive manner, the transcriptional profile of lipid sensitive mediators. The antioxidant potential in general, and total carotenoids in particular, were improved by the microalga, regardless lysozyme or Rovabio^®^.

**Conclusions:**

Summing up, *A. platensis*, individually and combined with feed enzymes, impacts negatively on piglets’ growth but improves the systemic antioxidant potential and changes plasma lipids with a minor modulation on related hepatic metabolic pathways.

## Background

In the pig industry, feed is a paramount topic. In the past two decades, there has been a major investment on the development of pig nutrition and on the improvement of meat quality to satisfy consumers’ demands. Cereal grains and soybean are the main energy and protein sources, respectively, for pig diets [1]. The high economic and environmental costs associated with production and transport of these ingredients over large distances and their direct competition with human consumption have important implications for the sustainability of feed and animal production [2]. Therefore, alternative sources of protein and well-balanced amino acids are urgently needed, as are sources of essential *n*-3 long-chain polyunsaturated fatty acids (LCPUFA), vitamins, minerals, carotenoids and bioactive compounds in animal feeding [3].

The use of microalgae in feed and food represents a promising strategy to solve this problem because microalgae are a natural resource with recognized beneficial health implications for both animals and humans [4]. Marine autotrophic microalgae bear attractive properties for sustainable animal production [5]. Although the nutritional profiles of microalgae differ substantially with the species, the majority is characterized by protein, carbohydrate, and lipid contents that are comparable, if not superior, to conventional feedstuffs [6]. In line with this, *Arthrospira* is a genus of these microalgae, characterized by cylindrical, multicellular trichomes in an open left-hand helix (reviewed by Madeira et al. [6]). *Arthrospira platensis* in particular, formerly known as Spirulina, is a rich source of organic nutrients with balanced content of vitamins, minerals, amino acids [7] and essential PUFA [8], as well as carotenoids and chlorophyll pigments with known antioxidants activity [9]. However, the microalga cell wall is recalcitrant, with a limited digestion and use by monogastrics [10].

Besides being poorly understood, the microalga cell wall has rigid components embedded within a plastic polymeric matrix, containing cellulose and, in some species, an additional tri-laminar sheath with algaenan, which is a compound that confers resistance to enzymatic degradation [11, 12]. In this respect, Carbohydrate-Active enZymes (CAZymes) that lyse the complex polysaccharides of the cell wall may be advantageous in the feed industry to improve nutrient utilization of microalgae [13]. In this respect, lysozyme is an enzyme that cleaves the peptidoglycan of prokaryote cell walls [14], thus promoting a better exposure of proteins and pigments to the endogenous repertoire of digestive enzymes [15]. Also, a commercial mixture of carbohydrate-degrading enzymes, like Rovabio^®^, can improve the profitable utilization of feed ingredients [16].

In spite of being sustainable alternatives to conventional ingredients for animal feeding, the effect of microalgae on the hepatic metabolism and redox status of monogastric species is currently too limited. In particular, the information available on the pattern of genes encoding for key lipogenic and lipolytic enzymes and associated transcription factors is urgently needed because these factors determine the rates of de novo fatty acid biosynthesis, fat uptake from blood and transport of fatty acids and lipid degradation [17]. This knowledge could help to improve the feeding strategies of pigs to address the swine industry needs and consumers’ demands. In line with this, we hypothesized that high levels of *A. platensis* incorporation in the diet, likely in association with exogenous CAZymes (lysozyme or Rovabio^®^), improve the antioxidant potential and change lipid metabolism in pigs, through the modulation of hepatic related metabolic pathways.

## Results

### Growth performance parameters

Data on piglets’ growth performance are shown in Table 1. Piglets fed *A. platensis* had lower final body weight (*p* = 0.009) and average daily gain (ADG) (*p* = 0.01) than piglets fed the control diet, but a higher feed conversion ratio (FCR) (*p* < 0.001). The average daily feed intake (ADFI) was not affected by dietary treatments (*p* > 0.05).

**Table 1:** Effect of *Arthrospira platensis,* individually or combined with exogenous CAZymes, on growth performance parameters of piglets

	Diets					
	Control	AP	AP + R	AP + L	SEM	*p*-value
Initial weight (kg)	12.1	11.7	12.1	11.9	0.15	0.808
Final weight (kg)	31.0^b^	28.3^a^	28.4^a^	27.8^a^	0.40	0.009
ADFI (g)	997	960	943	960	12.8	0.521
ADG (g)	677^a^	593^b^	582^b^	567^b^	12.4	0.001
FCR	1.48^a^	1.62^b^	1.62^b^	1.69^b^	0.023	< 0.001

Dietary treatments: cereal and soybean meal-based diet (control); basal diet with 10% of *Arthrospira platensis* (AP);

basal diet with 10% of *Arthrospira platensis* supplemented with 0.005% of Rovabio^®^ (AP + R); basal diet with 10% of

*Arthrospira platensis* supplemented with 0.01% of lysozyme (AP + L). *ADFI* average daily feed intake, *ADG* average daily weight gain, *FCR* feed conversion ratio

### Plasma biochemical profile

Plasma metabolites of piglets fed *A. platensis*, alone or combined with feed enzymes, are presented in Table 2. Total lipids (*p* = 0.011), total cholesterol (*p* < 0.001) and LDL-cholesterol (*p* < 0.001) were increased in piglets fed *A. platensis* individually. Piglets fed AP + R had higher HDL-cholesterol levels (*p* < 0.001) than the ones fed AP + L and control diets. These changes resulted in a lower total cholesterol: HDLcholesterol ratio in the AP + R group in relation to AP (*p* = 0.033). AP diet increased TAG (*p* < 0.001) when compared to AP + R. Total protein was lower (*p* < 0.001) in piglets fed *A. platensis* individually when compared to the other diets. AP + L increased the contents of glucose (*p* < 0.001) and creatinine (*p* < 0.001) relative to the other diets. Regarding the hepatic markers, *A. platensis* individually and combined with exogenous enzymes increased ALT (*p* < 0.001), while AST (*p* < 0.001) and ALP (*p* < 0.001) were increased in piglets fed the exogenous enzymes. GGT was decreased (*p* < 0.001) by *A. platensis* individually and combined with feed enzymes. Concerning the immunoglobulins, AP diet increased IgM (*p* < 0.001), whereas AP + L diet decreased IgG (*p* < 0.001) levels. In addition, AP + R diet decreased IgM concentrations (*p* < 0.001) when compared to the control diet.

**Table 2:** Effect of *Arthrospira platensis*, individually or combined with exogenous CAZymes, on plasma metabolites of piglets

	Diets					
	Control	AP	AP + R	AP + L	SEM	*p*-value
*Plasma metabolites*						
Total lipids (mg/L)1	3227^a^	3557^b^	3352^ab^	3466^ab^	69.0	0.011
TAG (mg/L)	433.3^ab^	496.7^b^	352.2^a^	524.0^b^	27.0	< 0.001
Total cholesterol (mg/L)	646.7^a^	780.0^b^	750.0^ab^	721.0^ab^	28.9	< 0.001
HDL-cholesterol (mg/L)	291.1^a^	337.8^bc^	358.9^c^	315.0^ab^	9.98	< 0.001
LDL-cholesterol (mg/L)	375.5^a^	466.7^b^	393.3^a^	417.0^ab^	18.4	0.009
VLDL-cholesterol (mg/L)2	86.7^ab^	99.3^b^	70.4^a^	104.8^b^	5.40	< 0.001
Total cholesterol/HDL-C	2.24^ab^	2.31^b^	2.08^a^	2.29^ab^	0.059	0.033
Glucose (mg/L)	1228^a^	1358^b^	1278^ab^	1525^c^	298	< 0.001
Urea (mg/L)	153.3	167.8	164.4	182.0	7.32	0.058
Creatinine (mg/L)	8.77^ab^	8.52^a^	9.41^bc^	9.08^c^	0.100	< 0.001
Total protein (g/L)	50.8^b^	44.0^a^	48.9^b^	50.1^b^	5.03	< 0.001
*Plasma hepatic markers*						
ALT (U/L)	36.0^a^	47.2^b^	58.9^c^	47.0^b^	1.21	< 0.001
AST (U/L)	40.4^a^	59.2^ab^	63.9^bc^	81.8^c^	5.08	< 0.001
ALP (U/L)	194.9^a^	213.4^a^	241.2^b^	266.6^b^	7.19	< 0.001
GGT (U/L)	42.2^b^	24.2^a^	23.4^a^	27.9^a^	1.47	< 0.001
*Immunoglobulins*						
IgA (mg/L)	23.3	26.7	24.4	25.0	2.23	0.761
IgG (mg/L)	1899^b^	2003^b^	1921^b^	1400^a^	95.4	< 0.001
IgM (mg/L)	484.4^b^	578.9^c^	336.7^a^	458.0^b^	14.6	< 0.001

Dietary treatments: cereal and soybean meal-based diet (control); basal diet with 10% of *Arthrospira platensis* (AP); basal diet with 10% of *Arthrospira platensis* supplemented with 0.005% of Rovabio^®^ (AP + R); basal diet with 10% of *Arthrospira platensis* supplemented with 0.01% of lysozyme (AP + L). ^a,b,c^Mean values within a row with unlike superscript letters are significantly different (*p* < 0·05)

ALT, alanine aminotransferase (EC 2.6.1.2); AST, aspartate aminotransferase (E.C. 2.6.1.1); ALP, alkaline phosphatase (EC 3.1.3.1); GGT, gamma-glutamyltransferase (EC 2.3.2.13)

^1^Total lipids = [total cholesterol] × 1.12 + [TAG] × 1.33 + 148

^2^VLDL-cholesterol = 1/5 [TAG]

### Plasma antioxidant potential

**Fig. 1:**
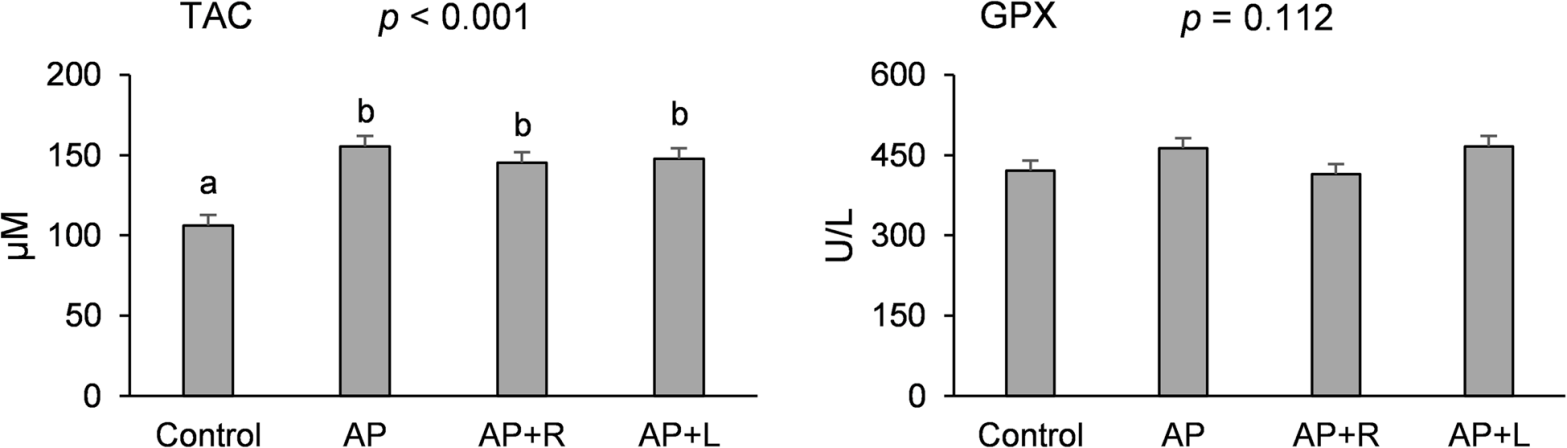
Effect of *Arthrospira platensis,* individually or combined with exogenous CAZymes, on plasma total antioxidant capacity (TAC) and glutathione peroxidase (GPX) activity. One unit of GPX is the amount of GPX that produces 1 μmol of GS-SG *per* min at pH = 7.6 and room temperature. Dietary treatments: cereal and soybean meal-based diet (control); basal diet with 10% of *Arthrospira platensis* (AP); basal diet with 10% of *Arthrospira platensis* supplemented with 0.005% of Rovabio^®^ (AP + R); basal diet with 10% of *Arthrospira platensis* supplemented with 0.01% of lysozyme (AP + L). ^a,b^Mean values with unlike letters are significantly different (*p* < 0·05)

The variations on plasma total antioxidant capacity (TAC) and glutathione peroxidase (GPX) activity from piglets fed *A. platensis* with or without CAZymes are presented in Fig. 1. *A. platensis* individually and combined with exogenous enzymes increased TAC levels (*p* < 0.001) when compared to the control diet. GPX remained unchanged by dietary treatments (*p* = 0.112).

### Hepatic total lipids and fatty acid composition

Hepatic lipid content and fatty acid composition of piglets fed *A. platensis*, individually or in combination with exogenous CAZymes, are presented in Table 3. Total lipid content (*p* = 0.977) and cholesterol (*p* = 0.737) were not affected by dietary treatments. The predominant fatty acids found in liver were: 18:0 (29.1–31.1%), 18:2*n*-6 (16.5–17.8%), 16:0 (13.5–15.3%), 20:4*n*-6 (13.2–14.9%) and 18:1*c*9 (10–11.7% of total FAME). The dietary treatments affected 8 out of 27 fatty acids identified. The proportion of 10:0 (*p* = 0.027) was higher in piglets fed AP + L diet when compared to the control diet. AP + R diet increased 16:0 (*p* = 0.021) when compared to the control diet. Also, 17:0 (*p* = 0.016) increased with AP and AP + L diets when compared to the control diet. AP + R and AP + L diets increased 18:3*n*-6 (*p* = 0.001) relative to the control diet. In contrast, piglets fed AP and AP + R diets had lower 20:2*n*-6 (*p* = 0.006) and 22:6*n*-3 (*p* = 0.005) when compared to piglets fed the control diet. The proportion of 20:5*n*-3 (*p* = 0.002) decreased in piglets fed *A. platensis* individually and combined with feed enzymes. Regarding the fatty acid sums and ratios, *A. platensis* alone and in combination with feed CAZymes increased SFA (*p* < 0.001) but decreased PUFA:SFA ratio (*p* < 0.001) (Table 3).

**Table 3:** Effect of *Arthrospira platensis,* individually or combined with exogenous CAZymes, on total lipids (g/100 g liver), cholesterol (mg/g), fatty acid composition (% total fatty acids), partial sums of fatty acids and related ratios in piglets’ liver

	Diets				SEM	*p*-value
	Control	AP	AP + R	AP + L		
Total lipids	2.37	2.37	2.41	2.42	0.104	0.977
Cholesterol	1.61	1.53	1.51	1.61	0.074	0.737
*Fatty acid composition*						
10:0	0.011^a^	0.014^ab^	0.017^ab^	0.025^b^	0.003	0.027
12:0	0.009	0.013	0.014	0.015	0.001	0.058
14:0	0.172	0.200	0.261	0.235	0.023	0.064
15:0	0.142	0.155	0.141	0.162	0.012	0.589
16:0	13.5^a^	13.9^ab^	15.3^b^	14.9^ab^	0.429	0.021
16:1*c*7	0.344	0.397	0.429	0.375	0.024	0.147
16:1*c*9	0.446	0.429	0.642	0.488	0.064	0.096
17:0	1.330^a^	1.703^b^	1.476^ab^	1.672^b^	0.088	0.016
17:1*c*9	0.153	0.140	0.166	0.135	0.012	0.301
18:0	29.1	31.1	29.7	30.7	0.945	0.415
18:1*c*9	10.2	10.0	11.7	10.1	0.516	0.093
18:1*c*11	1.520	1.467	1.625	1.591	0.049	0.121
18:2*n*-6	17.8	16.5	16.6	16.7	0.454	0.163
18:3*n*-6	0.162^a^	0.231^ab^	0.265^b^	0.253^b^	0.018	0.001
18:3*n*-3	0.410	0.361	0.429	0.473	0.038	0.220
18:4*n*-3	0.020	0.019	0.017	0.024	0.002	0.141
20:0	0.052	0.059	0.053	0.055	0.004	0.743
20:1*c*11	0.151	0.117	0.125	0.124	0.010	0.121
20:2*n*-6	0.613^b^	0.472^a^	0.453^a^	0.505^ab^	0.032	0.006
20:3*n*-6	0.715	0.771	0.810	0.741	0.055	0.647
20:4*n*-6	14.9	14.7	13.2	13.7	0.758	0.332
20:3*n*-3	0.167	0.157	0.151	0.178	0.014	0.526
20:5*n*-3	0.517^b^	0.362^a^	0.332^a^	0.353^a^	0.035	0.002
22:0	0.033	0.044	0.037	0.033	0.003	0.077
22:1*n*-9	0.326	0.489	0.502	0.544	0.072	0.189
22:5*n*-3	1.843^b^	1.493^ab^	1.356^ab^	1.296^a^	0.138	0.034
22:6*n*-3	1.752^b^	1.164^a^	1.016^a^	1.326^ab^	0.140	0.005
Others	3.574	3.425	3.217	3.276	0.175	0.477
*Fatty acid partial sums*						
SFA^1^	14.3^a^	26.2^b^	38.0^c^	55.7^d^	2.069	< 0.001
MUFA^2^	43.0	45.5	45.5	46.1	1.034	0.169
PUFA^3^	47.4	45.4	45.6	44.7	1.096	0.356
*n*-3 PUFA^4^	17.8	17.5	15.9	16.4	0.813	0.327
*n*-6 PUFA^5^	29.5	27.9	29.6	28.2	0.676	0.193
*Fatty acid ratios*						
PUFA:SFA	4.420^b^	1.817^a^	1.236^a^	0.804^a^	0.510	< 0.001
*n*-6:*n*-3	1.664	1.628	1.895	1.774	0.098	0.229

Dietary treatments: cereal and soybean meal-based diet (control); basal diet with 10% of *Arthrospira platensis* (AP); basal diet with 10% of *Arthrospira platensis* supplemented with 0.005% of Rovabio^®^ (AP + R); basal diet with 10% of *Arthrospira platensis* supplemented with 0.01% of lysozyme (AP + L)

^a,b,c^Mean values within a row with unlike superscript letters are significantly different (*p* < 0·05)

^1^SFA = 10:0 + 12:0 + 14:0 + 15:0 + 16:0 + 17:0 + 18:0 + 20:0 + 22:0

^2^MUFA = 16:1*c*7 + 16:1*c*9 + 17:1*c*9 + 18:1*c*9 + 18:1*c*11 + 20:1*c*11 + 22:1*n*-9

^3^PUFA = 18:2*n*-6 + 18:3*n*-6 + 18:3*n*-3 + 20:2*n*-6 + 20:3*n*-6 + 20:4*n*-6 + 20:3*n*-3 + 20:5*n*-3 + 22:5*n*-3 + 22:6*n*-3

^4^*n*-3 PUFA = 18:3*n*-3 + 20:3*n*-3 + 20:5*n*-3 + 22:5*n*-3 + 22:6*n*-3

^5^*n*-6 PUFA = 18:2*n*-6 + 18:3*n*-6 + 20:2*n*-6 + 20:3*n*-6 + 20:4*n*-6

### Hepatic tocopherols and pigments

The effect of *A. platensis* individually or combined with exogenous CAZymes on hepatic vitamin E compounds and pigments are presented in Table 4. α- and γ-tocopherols were affected by diets, being consistently decreased in piglets fed *A. platensis* with and without exogenous CAZymes (*p* < 0.001 and *p* = 0.0003, respectively). Conversely, piglets fed AP, AP + R and AP + L had higher total carotenoids (*p* < 0.001) than piglets fed the control diet.

**Table 4:** Effect of *Arthrospira platensis*, individually or combined with exogenous CAZymes, on α-tocopherol (μg/g), γ-tocopherol (μg/g) and pigments (μg/g) in piglets’ liver

	Diets				SEM	*p*-value
	Control	AP	AP + R	AP + L		
α-Tocopherol	1.64^b^	1.02^a^	1.16^a^	1.07^a^	0.086	< 0.001
γ-Tocopherol	0.073^b^	0.054^a^	0.050^a^	0.052^a^	0.004	0.0003
Chlorophyll-a^1^	0.360	0.780	0.469	0.580	0.202	0.510
Chlorophyll-b^2^	1.21	1.52	1.18	1.33	0.506	0.964
Total chlorophylls^3^	1.57	2.30	1.65	1.91	0.703	0.884
Total carotenoids^4^	0.660^a^	1.23^b^	1.28^b^	1.19^b^	0.083	< 0.001
Total chlorophylls and total Carotenoids^5^	2.23	3.52	2.93	3.10	0.741	0.666

Dietary treatments: cereal and soybean meal-based diet (control); based diet with 10% of *Arthrospira platensis* (AP); basal diet with 10% of *Arthrospira platensis* supplemented with 0.005% of Rovabio^®^ (AP + R); basal diet with 10% of *Arthrospira platensis* supplemented with 0.01% of lysozyme (AP + L)

^a,b^Mean values within a row with unlike superscript letters are significantly different (*p* < 0·05)

^1^Chlorophyll-a = 11.24 × A662 nm - 2.04 × A645 nm

^2^Chlorophyll-b = 20.13 × A645 nm - 4.19 × A662 nm

^3^Total chlorophylls (Ca + b) = 7.05 × A662 nm + 18.09 × A645 nm

^4^Total carotenoids (Cx + c) = (1000 × A470 nm - 1.90 × Ca - 63.14 × Cb) /214

^5^Total chlorophylls and carotenoids = (Ca + b) + (Cx + c)

### Gene expression levels of antioxidant enzymes and lipid metabolism players in the liver

The expression level of 8 genes controlling redox balance and 18 genes regulating lipid metabolism in piglets’ liver upon dependence of *A. platensis*, with or without feed enzymes, are presented in Table 5. For the antioxidant potential, only the transcriptional profile of nitric oxide synthase 2 (*NOS2*) (*p* = 0.048) was affected by diet, with higher mRNA levels found in piglets fed AP + R diet when compared to piglets fed AP diet. In turn, the dietary treatments affected 4 out of 18 key lipogenic enzymes and associated transcription factors. The AP + R and AP + L diets down-regulated the relative expression level of acetyl-CoA carboxylase α (*ACACA*) (*p* = 0.044) when compared to the control diet. AP + L diet upregulated the relative expression level of carnitine palmitoyltransferase 1A (*CPT1A*) (*p* = 0.037) when compared to AP diet, and down-regulated the relative expression level of fatty acid desaturase 2 (*FADS2*) (*p* = 0.028) when compared to the control diet. Also, AP + R decreased mRNA levels of fatty acid binding protein 1 (*FABP1*) (*p* = 0.049) relative to the control diet.

**Table 5:** Effect of *Arthrospira platensis*, individually or combined with exogenous CAZymes, on gene expression levels (relative mRNA level) in piglets’ liver

	Diets				SEM	*p*-value
	Control	AP	AP + R	AP + L		
Antioxidant potential						
*CAT*	37.92	37.95	29.39	30.91	3.139	0.116
*GPX1*	2.577	1.416	1.958	2.210	0.436	0.305
*GSR*	0.213	0.206	0.190	0.220	0.021	0.779
*SOD1*	7.048	6.357	5.543	5.831	0.439	0.097
*SOD2*	0.258	0.222	0.223	0.202	0.025	0.433
*SOD3*	0.073	0.073	0.091	0.094	0.011	0.389
Vasodilation						
*NOS2*	0.001^ab^	0.0007^a^	0.0024^b^	0.0012^ab^	0.0004	0.048
*NOS3*	0.287	0.405	0.287	0.299	0.082	0.693
Lipid metabolism						
*ACACA*	0.414^b^	0.354^ab^	0.261^a^	0.229^a^	0.050	0.044
*APOA5*	4.459	3.581	3.999	4.183	0.782	0.879
*CEBPA*	0.024	0.030	0.028	0.033	0.003	0.215
*CHREBP*	1.072	1.107	1.368	1.346	0.142	0.318
*CPT1A*	0.384^ab^	0.369^a^	0.421^ab^	0.564^b^	0.052	0.037
*CRAT*	0.970	0.804	0.921	0.821	0.091	0.511
*DGAT*	0.277	0.286	0295	0.279	0.018	0.907
*FABP1*	19.7^b^	13.4^ab^	10.5^a^	14.1^ab^	2.323	0.049
*FADS1*	4.863	4.981	3.386	2.705	0.850	0.164
*FADS2*	4.637^b^	3.919^ab^	2.966^ab^	2.513^a^	0.524	0.028
*FASN*	0.513	0.438	0.545	0.622	0.134	0.795
*HSL*	0.018	0.018	0.021	0.025	0.003	0.338
*LPIN1*	0.036	0.036	0.037	0.033	0.006	0.969
*PFKL*	0.254	0.243	0.273	0.269	0.020	0.712
*PLIN2*	0.038	0.034	0.049	0.032	0.009	0.609
*PPARA*	2.011	2.206	1.947	2.376	0.258	0.620
*SCD*	11.20	6.900	4.863	4.488	2.605	0.252
*SREBF1*	6.435	6.470	6.028	4.766	1.233	0.718

Dietary treatments: cereal and soybean meal-based diet (control); basal diet with 10% of *Arthrospira platensis* (AP); basal diet with 10% of *Arthrospira platensis* supplemented with 0.005% of Rovabio^®^ (AP + R); basal diet with 10% of *Arthrospira platensis* supplemented with 0.01% of lysozyme (AP + L)

^a,b^Mean values within a row with unlike superscript letters are significantly different (*p* < 0·05)

### Principal component analysis

**Fig. 2:**
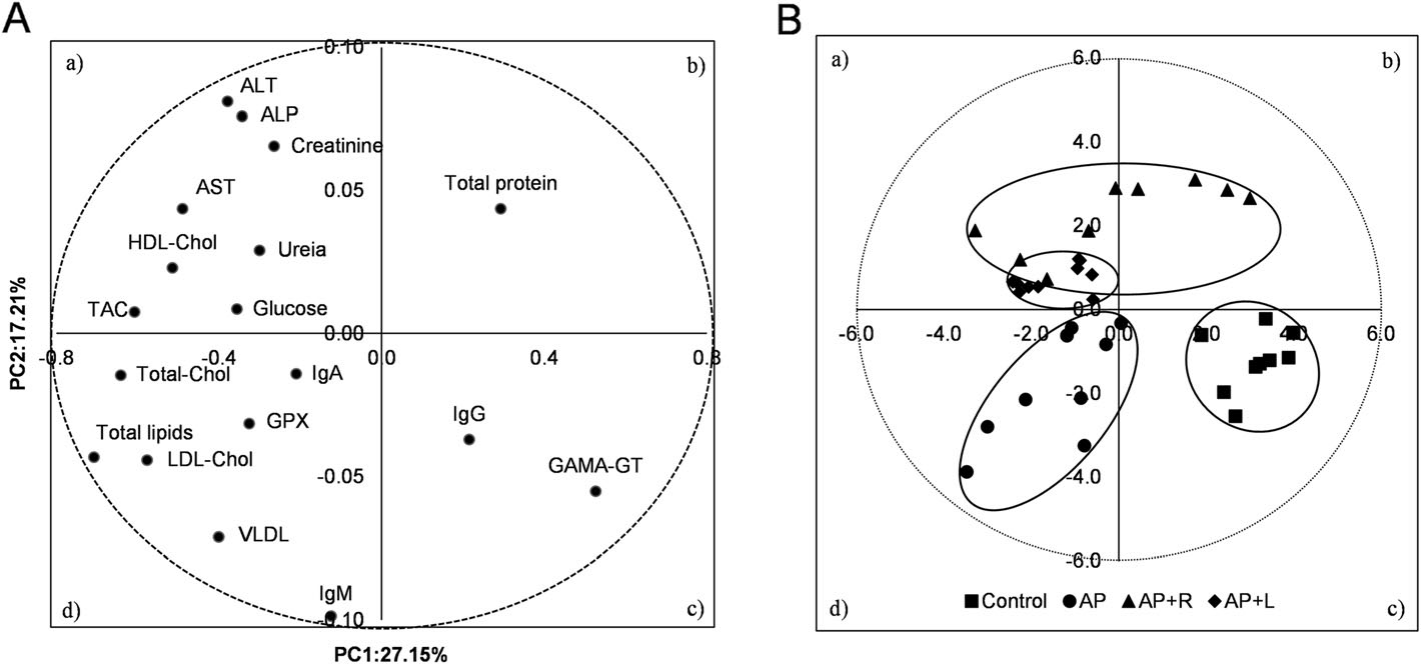
Loading plot of the first and second principal components (PC) of the pooled data **a** and component score vectors **b** using plasma metabolites from piglets fed *Arthrospira platensis*, individually or combined with exogenous CAZymes. Dietary treatments: cereal and soybean meal-based diet (control); basal diet with 10% of *Arthrospira platensis* (AP); basal diet with 10% of *Arthrospira platensis* supplemented with 0.005% of Rovabio^®^ (AP + R); basal diet with 10% of *Arthrospira platensis* supplemented with 0.01% of lysozyme (AP + L)

A principal component analysis (PCA) was performed with all data. It was verified that fatty acid composition, cholesterol, α- and γ-tocopherols, total pigments and gene expression levels in the liver had no relationship using this discriminant analysis. As so, a PCA is presented using only the plasma metabolites to describe the variability of the pooled data into two dimensions (Fig. 2 (a)). The score plot of the first two PC explained 44.3% of the total variability, with 27.1% for PC1 and 17.2% for PC2 (Table 6). The PC1 was characterized by variables with positive loadings, such as GGT and IgG, and by variables with negative loadings, such as total lipids, total cholesterol, TAC, LDL-cholesterol, HDL-cholesterol, AST, VLDL-cholesterol, TAG, ALT, glucose, ALP, GPX, urea, total protein, creatinine, IgA and IgM (Table 6). Concerning the PC2, all variables had small contributions with loadings varying between − 0.10 and 0.08.

**Table 6:** Loadings for the first two principal components (PC)

Variables	PC1	PC2
Total lipids	−0.71	−0.04
TAG	−0.40	−0.07
Total Cholesterol	−0.64	−0.01
HDL-cholesterol	−0.51	0.02
LDL-cholesterol	−0.58	−0.04
VLDL-cholesterol	−0.40	−0.07
Glucose	−0.36	0.01
Urea	−0.30	0.03
Creatinine	−0.27	0.07
Total protein	0.29	0.04
ALT	−0.38	0.08
AST	−0.49	0.04
ALP	−0.34	0.08
GGT	0.53	−0.05
IgA	−0.21	−0.01
IgG	0.21	−0.04
IgM	−0.13	−0.10
TAC	−0.61	0.01
GPX	−0.33	−0.03

The score plot depicted in Fig. 2 (b) showed the location of the four experimental groups, control, AP, AP + R and AP + L, in the multivariate space of the first two PC. These scores were notably arranged into two clusters, corresponding to control and AP diets. The control diet was located in quadrant *d*, while AP diet was located in quadrant *c*. AP + L diet was confined to quadrant *a*. The AP + R diet was dispersed across quadrants *a* and *b*.

## Discussion

Herein we assessed, for the first time, the molecular mechanisms of hepatic lipid metabolism and antioxidant potential under the influence of *A. platensis* as feed ingredient, individually and combined with two exogenous CAZymes (lysozyme and a commercial mixture of carbohydrate-degrading enzymes, named Rovabio^®^). In fact, several studies report the use of Spirulina as supplement in piglets feeding [18–20], but not as an ingredient (> 1% in the diet).

Piglets fed diets with 10% of *A. platensis*, had lower ADG but higher FCR than piglets fed a control diet, regardless the addition of feed enzymes. These findings partially agree with the literature. In a general literature overview, the inclusion of *A. platensis* as a dietary supplement increases ADG but negatively affects FCR (reviewed by Madeira et al. [6]). However, ADFI was here unaffected by dietary treatments. Total tract apparent digestibility of crude protein was higher in the control group than in *A. platensis* fed groups [21]. Lower protein digestibility is associated with higher digesta viscosity, which limits the access of endogenous enzymes to their target substrates. The decrease observed in piglets’ performance was due to the low digestibility and gelation of *A. platensis* proteins in the intestine, as a direct consequence of their proteolytic resistance to the piglet endogenous peptidases [21]. Digestible energy reached higher values on piglets fed control and *A. platensis* combined with Rovabio^®^ diets, which are in agreement with piglets’ growth performance, as the obtained values were also higher in the control group [21]. In addition, crude fat digestibility increased in piglets fed *A. platensis* combined with Rovabio^®^ and lysozyme, when compared to the control group, which indicates that enzymes were effective in degrading *A. platensis* cell wall, thus facilitating the access of digestive enzymes to the cell content [21].

*A. platensis* has also been exploited for therapeutic purposes of various conditions [22], such as anaemia, hepatotoxicity, reduction of cholesterol and prevention of cardiovascular diseases, and hyperglycaemia [23, 24]. Although the plasma lipid profile was largely affected by diet, our data are not in line with the former reports. Total lipids, total cholesterol and LDL-cholesterol were higher in piglets fed *A. platensis*. In fact, total cholesterol exceeded the reference values [25] in piglets from all dietary treatments. *A. platensis* is known for positive effects on cholesterol metabolism by increasing HDL, which can lead to healthy cardiovascular functions [26, 27]. This effect was confirmed by our data only when this microalga was combined with commercial Rovabio^®^. The increment of “bad cholesterol” promoted by *A. platensis* was countering by reverse cholesterol transport of HDL, decreasing the ratio total cholesterol: HDL-cholesterol and thus mitigating cardiovascular risk factors [28]. Additional discrepancies between our results and literature might be explained by the use of distinct dietary levels and experimental animal models, such as rodents and rabbits.

For hepatic markers, ALT activity was higher with *A. platensis*, and even more with supplementation of both exogenous CAZymes. In line with this, AST and ALP were also higher in piglets fed *A. platensis* combined with Rovabio^®^ and lysozyme. Contrarily, the GGT activity was lower in piglets fed *A. platensis*, with and without exogenous CAZymes. All in all, these variations on the hepatic function are devoid of clinical relevance because the levels of enzymatic activity found are still within the reference figures for pigs (31–58 for ALT, 32–84 for AST and 10–52 U/L for GGT, respectively [25]). If urea variations reflect unaffected renal function, creatinine reached the highest values with lysozyme and Rovabio^®^. Glucose was found increased with *A. platensis* incorporation, alone and combined with lysozyme, but this increase was apparently mitigated by Rovabio®^®^, suggesting a positive effect of the commercial mixture of carbohydrate-degrading enzymes on glycemia homeostasis.

It has been reported that *A. platensis* improves the immune system [29] and exhibits anti-inflammatory properties [30, 31]. While individually *A. platensis* increased IgM levels, its combination with lysozyme decreased IgG concentrations, reinforcing *A. platensis* ability for modulating some immune responses.

Total antioxidant capacity is a marker of global antioxidant defence, used as an accurate assessment of redox status in vivo [32]. *A. platensis*, with and without commercial enzymes, increased TAC in plasma, which is consistent with hepatic total carotenoids increase, rather than with non-variations of GPX activity. *A. platensis* contains a variety of natural carotene and xanthophyll phytopigments, which turns this microalga into a good nutritional supplement for human and animal feed [33]. GPX plays an important role in protecting haemoglobin, red blood cell enzyme activity and biological cell membranes against oxidative damage [34] and its activity reaches the highest values in the liver and erythrocytes [35]. Herein, the enzymatic activity of GPX measured in plasma had no changes across dietary treatments, which is consistent with similar transcriptional profile found in the liver. The gene expression levels were higher for catalase (*CAT*), superoxide dismutase 1 (*SOD1*) and glutathione peroxidase 1 (*GPX1*), in this particular order. However, none of these genes was affected by dietary treatments in the liver. Some studies have shown that weaning systematically decreases the antioxidant potential and increases the generation of free radicals in tissues and blood [36, 37]. SOD and CAT enzymes constitute the first line of antioxidant defence in the body [38], being the values found for their relative gene expression, in accordance with the literature. Other important antioxidants, some of them with extracellular origin, in particular vitamin E, might have contributed to improve redox status in piglets fed *A. platensis.* Curiously, data on α- and γ-tocopherol contents were observed in the opposite direction. The values found for vitamin E tocopherols in the liver were lower in piglets fed the microalga and the microalga plus exogenous CAZymes and do not match the original amounts on diets formulation, suggesting that feeding *A. platensis* at this high level of incorporation reduces vitamin E, through mechanisms that warrant further elucidation. Nitric oxide, a free radical that acts as a biological mediator in several processes, including neurotransmission as well as antimicrobial and antitumoral activities, is catalysed by the conversion of L-arginine to nitric oxide by nitric oxide synthase (NOS). Only NOS2, a vasodilator marker, was affected by diets, being its gene up-regulated by *A. platensis* in combination with the commercial Rovabio^®^. Neuronal NOS, endothelial NOS and inducible NOS [39] are expressed in the liver and activated by a combination of lipopolysaccharide (LPS) and certain cytokines, mostly common associated with the weaning process. Early weaning predisposes the pig intestine to structural and functional alterations, due to the increase in *Escherichia coli* populations. These bacteria use the LPS derived from their cell wall as an important pathogenic factor [40].

Liver is the principal site of cholesterol synthesis and fatty acid oxidation, whereas de novo lipogenesis occurs essentially in both liver and adipose tissue [41]. The majority of individual fatty acids quantified in the liver were not affected by the microalga nor by the exogenous CAZymes. This result aligns well with the low fatty acids content of *A. platensis* [6]. Nevertheless, the sum of SFA increased in piglets fed diets containing the microalga and exogenous CAZymes and, consequently, PUFA:SFA ratio decreased. For lipid metabolism, higher gene expression levels were found for: apolipoprotein A-V (*APOA5*) *>* fatty acid binding protein 1 (*FABP1*) *>* fatty acid desaturase 1 (*FADS1*) *> FADS2 >* peroxisome proliferator-activated receptor alpha (*PPARA*) > stearoyl-CoA desaturase (*SCD*) > sterol regulatory element binding transcription factor 1 (*SREBF1*). *A. platensis* with exogenous CAZymes down-regulated *ACACA*, a key lipogenic enzyme for fatty acid biosynthesis, together with fatty acid synthase (*FASN*), and *SCD* or delta9 desaturase [36] that remained unchanged across dietary treatments, therefore validating the similar values of total lipids observed in the liver. *FADS1*, encoding for ∆5 desaturase, and *FADS2*, encoding for ∆6 desaturase, are membrane-bound enzymes that catalyse the synthesis of PUFA [42]. The mRNA levels of *FADS2* were decreased in piglets fed *A. platensis* with lysozyme, not accompanied by a decrease in PUFA content [43]. *FADS1* was not affected by dietary treatments although it showed identical gene expression magnitude as *FADS2*, which might be explained by the fact that *FADS2* is sensitive to lysozyme. *FABP1* prevents lipotoxicity of free fatty acids and regulates fatty acid trafficking and partition [44]. Its relative gene expression level was decreased by *A. platensis* combined with Rovabio^®^. This finding requires further investigation. The mRNA levels of carnitine O-acetyltransferase (*CRAT*), one of the enzymes responsible for fatty acid β oxidation [45], as well as *PPARA*, a major inducer of fatty acid oxidation that suppresses fat synthesis [46], were kept unchanged by both *A. platensis* and exogenous CAZymes.

## Conclusion

Under the experimental conditions tested in this study, *A. platensis* incorporated as feedstuff, supplemented or not with two exogenous CAZymes (lysozyme and commercial Rovabio^®^), impacted negatively on piglets’ growth and increased systemic lipemia, without changing the hepatic fatty acid content. In fact, dietary treatments had a minor effect on fatty acid composition and transcriptional profile of lipid sensitive mediators in the liver. By contrast, and validating our initial hypothesis, the addition of this microalga benefited the systemic redox balance, regardless the presence of lysozyme or Rovabio^®^, as shown by the clear discrimination between the control diet and *A. platensis* diet in the multidimensional space of the PCA analysis. However, this positive variation was not followed by up-regulation of the first line of antioxidant defence, *CAT*, *SOD* and *GPX* enzymes, or the level of vitamin E compounds in piglets’ liver. In contrast, these results are supported by total carotenoids increase, which are compounds known to counterbalance oxidative stress. In view of these resuls, further studies are encouraged to incorporate lower percentages of this microalga in pigs feed before final conclusions could be drawn.

## Methods

### Animals and experimental diets

All the procedures used were reviewed by the Ethics Commission of Instituto Superior de Agronomia (ISA) and approved by the Animal Care Committee of the National Veterinary Authority (Direção Geral de Alimentação e Veterinária, Portugal), following the European Union legislation (2010/63/EU Directive).

The experimental trial was conducted at the facilities of Instituto Superior de Agronomia (ISA), Universidade de Lisboa. Forty male *post*-weaned piglets from Large White × Landrace sows crossed with Pietrain boars, weaned at 28 days of age and with an initial live weight of 12.0 ± 0.89 kg were obtained with consent from a commercial farm. After an adaptation period of two days, piglets were evenly distributed into four homogeneous groups of 10 piglets each (calculation of sample size by power analysis) and randomly individually allocatedin pens (1.9 × 1.1 m), equipped with one stainless steel bowl drinker with nipple, one creep feeder and a modular plastic slatted floor. The room was environmentally controlled with air ventilation, as described by Correia et al. [47]. Piglets had ad libitum access to feed and water. Throughout the experiment, the supplied feed was recorded daily, whereas refusals and piglets were weighed weekly, just before feeding, in order to calculate ADFI, ADG and FCR. Each group received one of the four experimental diets: 1) cereal and soybean meal-based diet (control); 2) basal diet with 10% of *A. platensis* (AP); 3) basal diet with 10% of *A. platensis* supplemented with 0.005% of Rovabio^®^ Excel AP (Adisseo, Antony, France) (AP + R); 4) basal diet with 10% of *A. platensis* supplemented with 0.01% of lysozyme (62,971, Sigma-Aldrich Ltd., St. Louis, MO, USA) (AP + L). Freeze-dried *A. platensis* powder was obtained from Sopropeche (Wimille, France). Rovabio^®^ Excel AP was composed by endo–1,4-β-xilanase 22,000 viscosity units/g and endo-1,3(4)-β-glucanase 30,000 viscosity units/g. The ingredients and feed additives of the experimental diets are described in Table 7.

**Table 7:** Ingredients and detailed chemical composition of the experimental diets

	Diets			
	Control	AP	AP + R	AP + L
*Ingredients (g/kg, as fed basis)*				
Wheat	439	460	460	458
Corn	150	170	170	170
Soybean meal 48	250	110	110	110
Whey powder	100	100	100	100
Soybean oil	30	30	30	30
Spirulina	0	100	100	100
Rovabio^®^ Excel AP	–	–	0.05	–
Lysozyme	–	–	–	0.1
L-lysine	5	6	6	6
DL-methionine	1	1	1	1
L-threonine	1	–	–	–
Calcium carbonate	5	6	6	6
Dicalcium phosphate	13	12	12	12
Sodium chloride	3	2	2	2
Vitamin-mineral complex^1^	3	3	3	3
*Metabolizable energy (kcal/kg DM)*^*2*^	3.738	3.809	3.789	3.818
*Chemical composition (g/100 g, as fed basis)*				
DM	89.8	90.0	90.0	90.0
CP	17.9	18.1	17.9	17.8
NDF	14.6	11.9	11.9	11.8
ADF	4.21	3.97	4.06	3.94
Ash	5.11	4.76	4.89	4.75
Crude fat	5.28	5.62	5.87	5.80
*Fatty acid composition (% total fatty acids)*				
12:0	0.123	0.122	0.146	0.140
14:0	0.396	0.479	0.520	0.531
16:0	13.5	17.8	18.3	19.3
16:1*c*9	0.140	0.817	0.875	0.836
18:0	3.17	3.19	3.32	3.62
18:1*c*9	24.1	21.1	20.5	20.1
18:1*c*11	1.30	1.23	1.28	1.27
18:2*n*-6	48.6	43.1	42.0	39.1
18:3*n*-3	4.55	4.17	4.31	4.22
20:0	0.364	0.325	0.324	0.323
20:1*c*11	0.298	0.572	0.559	0.820
22:0	0.365	0.365	0.365	0.421
Pigments (μg/g)				
Chlorophyll-a^3^	2.70	108	112	132
Chlorophyll-b^4^	4.97	14.6	13.0	17.5
Total chlorophylls^5^	7.67	122	125	149
Total carotenoids^6^	2.41	11.6	12.6	13.0
Total chlorophylls and total carotenoids^7^	10.1	134	138	162
*Diterpene profile (μg/g)*				
β-Carotene	0.160	3.57	3.14	2.15
α-Tocopherol	7.41	12.0	12.3	12.9
β-Tocopherol	0.676	0.254	0.215	0.213
γ-Tocopherol	1.054	0.997	1.052	0.925
α-Tocotrienol	1.092	0.504	0.923	0.994

Dietary treatments: cereal and soybean meal-based diet (control); basal diet with 10% of *Arthrospira platensis* (AP); basal diet with 10% of *Arthrospira platensis* supplemented with 0.005% of Rovabio^®^ (AP + R); basal diet with 10% of *Arthrospira platensis* supplemented with 0.01% of lysozyme (AP + L)

^1^Premix provided *per* kg of complete diet: vitamin A, 6500 UI; vitamin D_3_, 1500 UI; vitamin E, 15 mg; vitamin K_3_, 1 mg; vitamin B_1_, 1 mg; vitamin B_2_, 3 mg; vitamin B_6_, 2 mg; vitamin B_12_, 0.02 mg; pantothenic acid, 10 mg; nicotinic acid, 15 mg; folic acid, 0.5 mg, biotin, 0.03 mg; betaine, 115 mg; vitamin C, 20 mg; Copper, 100 mg; iron, 100 mg; iodine, 0.5 mg; manganese 50 mg; selenium, 0.15 mg; zinc, 100 mg; butylated hydroxytoluene, 3 mg

^2^Metabolizable energy (kcal/kg DM) = 4412–11,06 × Ash (g/kg DM) + 3,37 × Crude Fat (g/kg DM) − 5,18 × ADF (g/kg DM) [63]

^3^Chlorophyll-a = 11.24 × A662 nm - 2.04 × A645 nm

^4^Chlorophyll-b = 20.13 × A645 nm - 4.19 × A662 nm

^5^Total chlorophylls (Ca + b) = 7.05 × A662 nm + 18.09 × A645 nm

^6^Total carotenoids (Cx + c) = (1000 × A470 nm - 1.90 × Ca - 63.14 × Cb)/214

^7^Total chlorophylls and carotenoids = (Ca + b) + (Cx + c)

Diets were analysed for dry matter, ash and crude protein (automated Kjeldahl method), CF, NDF and ADF contents, following AOAC [48] methods. Fatty acid methyl esters (FAME) of the experimental diets were analysed by one-step extraction and transesterification, using heneicosaenoic acid (21:0) methyl ester as the internal standard [49]. The pigments of diets were measured according to Teimouri et al. [50], with slight modifications. Briefly, the samples were extracted with acetone and stored under agitation overnight, then centrifuged at 4000 rpm for 5 min and measured by UV-Vis spectrophotometry (Ultrospec 3100 pro, Amersham Biosciences, Little Chalfont, UK). The pigment content was quantified according to Hynstova et al. [51]. The quantification of tocopherols and tocotrienols in the diets involved a direct saponification, a single *n*-hexane extraction and analysis of the extracted compounds by normal-phase HPLC using fluorescence detection (FD), as described by Prates et al. [52]. The chemical composition, fatty acids and pigments contents of the experimental diets are shown in Table 7.

### Slaughter and sampling

After an experimental period of 28 days, during which no sick or dead animals were recorded, piglets were slaughtered using electrical stunning followed by exsanguination, according to commercial abattoirs standard procedures. Blood was collected from the jugular vein and centrifuged at 1500 *g* for 15 min to obtain plasma. Samples for gene expression analysis were collected from the middle lobe of liver, rinsed with sterile RNAse-free cold saline solution, cut into small pieces, stabilized in RNA Later® solution (Qiagen, Hilden, Germany) and stored at − 80 °C. For fatty acid composition and pigments, liver samples were vacuum packed and stored at − 20 °C, until analysis.

### Plasma metabolites

Total cholesterol, HDL-cholesterol, LDL-cholesterol, triacylglycerols (TAG), phospholipids, total protein, urea, creatinine and glucose concentrations, aspartate aminotransferase (AST), alanine aminotransferase (ALT), alkaline phosphatase (ALP) and gamma-glutamyltransferase (GGT) were analysed in a Modular Hitachi Analytical System (Roche Diagnostics, Mannheim, Germany), through diagnostic kits (Roche Diagnostics). VLDL-cholesterol and total lipids were calculated, according to Friedewald et al. [53] and Covaci et al. [54] formulas, respectively. The immunoglobulins profile (IgA, IgG and IgM) was determined by immunoturbidimetry. Total antioxidant capacity was determined using the QuantiChromTM Antioxidant Assay Kit (DTAC-100, Bioassay Systems, Hayward, CA, USA). Glutathione peroxidase activity was determined using the EnzyChromTM Glutathione Peroxidase Assay Kit (EGPX-100, Bioassay Systems). One unit of GPX is the amount of GPX that produces 1 μmol of GS-SG *per* min at pH = 7.6 and room temperature.

### Hepatic lipid extraction and fatty acid composition

After liver samples lyophilisation (− 60 °C and 2.0 hPa), total lipids were extracted 2× and gravimetrically measured by the Folch et al. [55] method, using dichloromethane and methanol (2:1 v/v), as reported by Carlson [56]. Fatty acids were converted to methyl esters (FAME) by a combined transesterification procedure using NaOH in anhydrous methanol (0.5 M), followed by HCl:methanol (1:1 v/v), at 50 °C during 30 and 10 min, respectively, in accordance to Raes et al. [57]. FAME were determined using a gas chromatograph HP6890A (Hewlett–Packard, PA, USA), with a flame ionization detector (FID) and a CP-Sil 88 capillary column (100 m, 0.25 mm i.d., 0.20 μm film thickness; Chrompack, Varian Inc., Walnut Creek, CA, USA), using the conditions described in Alves & Bessa [58]. The quantification of total FAME was carried out using heneicosaenoic acid (21:0) as internal standard and on the conversion of relative peak areas into weight percentages. Fatty acids were identified according to their retention times, corresponding to their FAME standards from Supelco Inc. (Bellefonte, PA, USA) and expressed as g/100 g of total fatty acids.

### Determination of total cholesterol and diterpenes in the liver

The simultaneous analysis of total cholesterol and tocopherols in liver samples (0.75 g) was performed, according to Prates et al. [51]. After the direct saponification of samples, an aliquot of the *n*-hexane layer was filtered and injected into an HPLC system (Agilent 1100 Series, Agilent Technologies Inc., Palo Alto, CA, U.S.A.), using a normal-phase silica column (Zorbax RX-Sil, 250 mm × 4.6 mm i.d., 5 μm particle size, Agilent Technologies Inc., Palo Alto, CA, U.S.A.), with fluorescence detection of tocopherols (excitation wavelength of 295 nm and emission wavelength of 325 nm) and UV–visible photodiode array detection of cholesterol (202 nm). Total cholesterol and tocopherols contents were calculated 2×, based on the external standard technique from a standard curve of peak area vs. concentration.

### Determination of pigments in the liver

The contents of chlorophyll-a, chlorophyll-b and total carotenoids were measured following the procedure of Teimouri et al. [50], with minor modifications. For the pigment determination, 10 mL of acetone (Merck KGaA, Darmstadt, Germany) was added to 1 g of fresh meat or 0.5 g of feed, then incubated at room temperature and shaken in the dark overnight. After extraction, the samples were centrifuged at 1500 *g* for 5 min and measured using a UV-Vis spectrophotometer (Ultrospec 3100 pro, Amersham Biosciences, Little Chalfont, UK). All procedures associated with pigments extraction and analyses were carried out in dim light because pigments are photosensitive. The pigment content was calculated, according to Hynstova et al. [51].

### Hepatic RNA isolation and complementary DNA synthesis

Total hepatic RNA was extracted and purified using Trizol (Invitrogen, CA, USA) and RNeasy mini kit (Qiagen), respectively. Before running the RT-PCR, RNA samples were subjected to DNAse I (Qiagen) treatment. All procedures followed the manufacturer’s instructions, according to Madeira et al. [59]. The quantification of RNA was carried out using a spectrophotometer (Nanodrop ND-2000c, NanoDrop, Thermo Fisher Scientific, Willmington, DE, USA). The A260/280 ratios ranged between 1.9 and 2.1. The High-Capacity cDNA Reverse Transcription Kit (Applied Biosystems, Foster City, CA, USA) was applied for reverse transcription Each 20 μL RT reaction included 1 μg of DNase-treated total RNA template, 50 nM random RT Primer, 1 × RT buffer, 0.25 mM of each dNTP, 3.33 U μL-1 multiscribe reverse transcriptase and 0.25 U μL-1 RNase inhibitor, during 10 min for 25 °C, 120 min for 37 °C and 5 min for 85 °C. The cDNA obtained was separated into several aliquots and kept at − 20 °C, until analysis.

### Real-time quantitative PCR of hepatic genes

Primer3 (https://bioinfo.ut.ee/primer3-0.4.0/) and Primer Express Software v. 2.0 (Applied Biosystems) based on *Sus scrofa* sequences (www.ncbi.nlm.nih.gov) were used for gene specific intron-spanning primers design, as described by Madeira et al. [58]. The selected primers were acquired from NZYTech (Lisbon, Portugal) and matched only the sequence to which they were constructed. To guarantee maximum DNA polymerization efficiency, the amplicon length ranged between 71 and 138 bp. Prior qPCR experiments, a conventional PCR was performed for all genes to confirm the amplified fragments. To corroborate the amplification, the products of PCR were sequenced and homology searches were checked with Blast (www.ncbi.nlm.nih.gov/blast). GeNorm30 and NormFinder31 software packages were applied for the analysis of the expression level stability of housekeeping genes. The most stable pair internal controls for normalization were RPLP0 and RPL27 genes. The gene specific primer sequences used for RT-qPCR are shown in Table 8. The efficiency of PCR for each amplicon was calculated with the StepOnePlus PCR System software (Applied Biosystems), by amplifying 5× serial dilutions of pooled cDNA and run 3×. All primer sets exhibited an efficiency ranging from 90 to 110% and correlation coefficients were over 0.99. qPCR reactions were carried out using the MicroAmp Optical 96-well plates (Applied Biosystems) in a StepOnePlus thermocycler (Applied Biosystems) in standard cycling conditions. The 12.5 μL PCR reaction mixture included 6.25 μL of 2 × Power SYBR Green PCR Master Mix (Applied Biosystems), 160 nM of forward and reverse primers, and 2 μL of diluted cDNA as template. No transcription and no template samples were applied as controls. The primer specificity and the formation of primer-dimers were verified by melt curve analysis and agarose gel electrophoresis. All analyses were performed 2×, and the relative amounts for each target gene were calculated using the geometric mean of RPLP0/RPL27 as normaliser. The relative gene expression levels were calculated using the Livak & Schmittgen [60] method, corrected for variation in amplification efficiency, as proposed by Fleige et al. [61].

**Table 8:** Gene specific primer sequences used for RT-qPCR

Gene symbol	Full gene name	GenBank accession number	Forward primer	Reverse primer	Product size (bp)
*ACACA*	Acetyl-CoA carboxylase alpha	NM_001114269.1	ggccatcaaggacttcaacc	acgatgtaagcgccgaactt	120
*APOA5*	Apolipoprotein A-V	NM_001159308.1	agggaaaggcttctgggacta	tgtctttcagtctcgtgggctc	107
*CAT*	Catalase	NM_214301.2	agaggaaacgcctgtgtgag	ttgtccagaagagcctgaatg	133
*CEBPA*	CCAAT/enhancer binding protein (C/EBP) alpha	XM_003127015.2	ggccagcacacacacattaga	cccccaaagaagagaaccaag	71
*ChREBP*	MLX interacting protein-like	XM_003481002.2	tgacatgatccagcctgacc	gggggctcagagaagtttga	126
*CPT1A*	Carnitine palmitoyltransferase 1A	NM_001129805.1	cgattatccaccagccagac	caccccataaccatcgtcag	120
*CRAT*	Carnitine O-acetyltransferase	NM_001113047.1	ggcccaccgagcctacac	atggcgatggcgtaggag	138
*DGAT*	Diacylglycerol acyltransferase	NM_214051.1	caactaccgtggcatcctga	tagaaacagccgtgcattgc	67
*FABP1*	Fatty acid binding protein 1	NM_001004046.1	aacttctccggcaaataccaa	attctgcacgatttccgatg	129
*FADS1*	Fatty acid desaturase 1	NM_001113041.1	gtgggtggacttggcctg	gatgtgcatggggatgtggt	166
*FADS2*	Fatty acid desaturase 2	NM_001171750.1	gccttacaaccaccagcatga	aggccaagtccacccagtc	122
*FASN*	Fatty acid synthase	NM_001099930.1	acaccttcgtgctggcctac	atgtcggtgaactgctgcac	112
*GPX1*	Glutathione peroxidase 1	NM_214201.1	ggagatcctgaattgcctca	gataaacttggggtcggtca	181
*GSR*	Glutathione-disulfide reductase	XM_003483635.4	ggtgtgtgccaacaaagagg	aaccctgcagcagcatttcatca	77
*HSL*	Hormone sensitive lipase	397,583	tcgtggctcaactccttcct	gggtgtcctgtgtctcgg	190
*LPIN1*	Lipin 1	NM_001130734.1	aagtcgccgccctgtatttc	ttgtcgctggcctgttttgt	67
*NOS2*	Nitric oxide synthase 2	NM_001143690.1	cctggtgccctgctttgt	ctgccagaaactgcggaag	118
*NOS3*	Nitric oxide synthase 3	NM_214295.1	ggctgcatgacattgagagc	ctcgtcgcggtagagatggt	98
*PFKL*	Phosphofructokinase liver	100,621,757	gctcaaggaggacaccgact	cgccagcatcttcagcat	85
*PLIN2*	Perilipin 2	NM_214200.2	catgtccggtgctctcccta	cccagtcacagcccctttag	160
*PPARA*	Peroxisome proliferator-activated receptor alpha	NM_001044526.1	tttccctctttgtggctgct	ggggtggttggtctgcaag	128
*SCD*	Stearoyl-CoA desaturase	NM_213781.1	agccgagaagctggtgatgt	gaagaaaggtggcgacgaac	140
*SOD1*	Superoxide dismutase 1	NM_001190422.1	gctgtaccagrgcaggtcctc	cacagtggccacaccatctt	125
*SOD2*	Superoxide dismutase 2	NM_214127.2	gtggaggccacatcaatcat	ccgacagatacagcggtcaa	148
*SOD3*	Superoxide dismutase 3	NM_001078688.1	accagttcggggacctgag	ggcgaagttgccgaagtct	104
*SREBF1*	Sterol regulatory element binding transcription factor 1	NM_214157.1	gtgctggcggaggtctatgt	aggaagaagcgggtcagaaag	86
**Housekeeping genes**					
*RPLP0*	Ribosomal phosphoprotein large P0 subunit	NM_001098598.1	tccaggctttaggcatcacc	ggctcccactttgtctccag	95
*RPL27*	Ribosomal protein L27	NM_001097479.1	gtactccgtggatattg	aacttgaccttggcct	102

### Statistical analysis

Data were checked for normal distribution by Shapiro-Wilk test and for variance homogeneity by Chi-Square test. Data were analysed using the Generalized Linear Mixed (GLM) model of SAS program (SAS Institute Inc., Cary, NC) [62] considering the piglet as experimental unit. Significant multiple comparisons test was carried out using the PDIFF option adjusted with Tukey-Kramer to determine statistical differences among dietary treatments. The level of significance was set at *p* < 0.05. A principal component analysis (PCA) was performed with individual plasma metabolites, hepatic markers and immunoglobulins from piglets. The PRINCOMP procedure was applied to a data set of 40 samples and 18 variables to reduce the dimensionality of the data set and to describe the variability of data into two dimensions. After data normalization, the principal components were considered significant if they contributed more than 5% for the total variance.

## Data Availability

All data generated during this study are included in this published article. The datasets generated during the current study are available from the corresponding author on demand.

## Abbreviations

ACACA: Acetyl-CoA carboxylase
ADFI: Average daily feed intake
ADG: Average daily gain
ALT: Alanine aminotransferase
ALP: Alkaline phosphatase
APOA5: Apolipoprotein A-V
CAT: Catalase
AP: *Arthrospira platensis*
AST: Aspartate aminotransferase
CEBPA: CCAAT/enhancer binding protein alpha
ChREBP: Carbohydrate response element binding protein
CPT1A: Carnitine palmitoyltransferase 1A
CRAT: Carnitine O-acetyltransferase
DGAT: Diacylglycerol O-acyltransferase
FABP1: Fatty acid binding protein 1
FADS1: Fatty acid desaturase 1
FADS2: Fatty acid desaturase 2
FAME: Fatty acid methyl esters
FASN: Fatty acid synthase
FCR: Feed convertion ratio
GPX1: Glutathione-disulfide reductase
GGT: Gamma-glutamyltransferase
GPX: Glutathione peroxidase
HSL: Hormone sensitive lipase
LPIN1: Lipin 1
MUFA: Monounsaturated fatty acids
NOS2: Nitric oxide synthase 2
NOS3: Nitric oxide synthase 3
OAZ1: Ornithine decarboxylase antizyme 1
PFKL: Phosphofructokinase liver
PLIN2: Perilipin 2
PPARA: Peroxisome proliferator-activated receptor alpha
PUFA: Polyunsaturated fatty acids
RPLP0: Ribosomal protein large P0
RPL27: Ribosomal protein L27
SFA: Saturated fatty acids
SCD: Stearoyl-CoA desaturase
SOD1: Superoxide dismutase 1
SOD2: Superoxide dismutase 2
SOD3: Superoxide dismutase 3
SREBP1: Sterol regulatory element binding protein 1
TAC: Total antioxidant capacity

## References

[1] FAO (Food and Agriculture Organization of the United Nations). Protein sources for the animal feed industry. In: Proceedings from the expert consultation and workshop, 29 April-3 may 2002. Bangkok: FAO; 2004.

[2] Manceron S, Ben-Ari T, Dumas P (2014). Feeding proteins to livestock: global land use and food vs feed competition. OCL.

[3] Florou-Paneri P, Christaki E, Giannenas I, Bonos E, Skoufos I, Tsinas A, Tzora A, Peng J (2014). Alternative protein sources to soybean meal in pig diets. J Food Agric Environ.

[4] Calder P (2012). Mechanisms of action of (n-3) fatty acids. J Nutr.

[5] Lum K, Kim J, Lei X (2013). Dual potential of microalgae as a sustainable biofuel feedstock and animal feed. J Anim Sci Biotechnol.

[6] Madeira MS, Cardoso C, Lopes PA, Coelho D, Afonso C, Bandarra NM, Prates JAM (2017). Microalgae as feed ingredients for livestock production and meat quality: a review. Livest Sci.

[7] Simkus A, Martinavicius V, Kulpus J, Simkiene A, Knietkute N, Stankeviciene M (2008). The effect of microalgae *Spirulina platensis* on physiological processes and productivity of fattening pigs. Zhivotnovadni nauki.

[8] Peiretti PG, Meineri G (2011). Effects of diets with increasing levels of *Spirulina platensis* on the carcass characteristics, meat quality and fatty acid composition on growing rabbits. Livest Sci.

[9] Madhava C, Bhat VB, Kiranmai G, Reddy MN, Reddanna P, Madyastha KM (2000). Selective inhibition of cyclooxygenase-2 by C-phycocyanin, a biliprotein from Spirulina platensis. Biochem Biophys Res Commun.

[10] Tibbetts SM. The potential for ‘next-generation’, microalgae-based feed ingredients for salmonid aquaculture in context of the blue revolution. In: Jacob-Lopes E, Zepka LQ, Queiroz MI, editors. Microalgal Biotechnology. London: IntechOpen; 2018. p. 151–75.

[11] Popper ZA, Tuohy MG (2010). Beyond the green: understanding the evolutionary puzzle of plant and algal cell walls. Plant Physiol.

[12] Gerken HG, Donohoe B, Knoshaug EP (2012). Enzymatic cell wall degradation of *Chlorella vulgaris* and other microalgae for biofuels production. Plant..

[13] Sander K, Murthy GS. Enzymatic degradation of microalgal cell walls. In American Society of Agricultural and Biological Engineers (ASEBE) Annual International Meeting 2009, 21–24 June 2009, Reno, Nevada.

[14] Oliver WT, Wells JE (2015). Lysozyme as an alternative to growth promoting antibiotics in swine production. J Anim Sci Biotechnol.

[15] Al-Zuhair S, Ashraf S, Hisaindee S, Darmaki NA, Battah S, Svistunenko D, Reeder B, Stanway G, Chaudhary A (2017). Enzymatic pre-treatment of microalgae cells for enhanced extraction of proteins. Eng Life Sci.

[16] Gunawardana P, Roland DA, Bryant MM (2009). Effect of dietary energy, protein, and a versatile enzyme on hen performance, egg solids, egg composition, and egg quality of Hy-line W-36 hens during second cycle, phase two. J Appl Poult Res.

[17] Zhao S, Wang J, Song X, Zhang X, Ge C, Gao S (2010). Impact of dietary protein on lipid metabolism-related gene expression in porcine adipose tissue. Nutr Metab (Lond).

[18] Grinstead GS, Tokach MD, Dritz SS, Goodband RD, Nelssen JL (2000). Effects of *Spirulina platensis* on growth performance of weanling pigs. Anim Feed Sci Technol.

[19] Nedeva R, Jordanova G, Kistanova E, Shumkov K, Georgiev B, Abadgieva D, Kacheva D, Shimkus A, Shimkine A (2014). Effect of the addition of *Spirulina platensis* on the productivity and some blood parameters on growing pigs. Bul J Agric Sci.

[20] Furbeyre H, Milgen J, Mene T, Gloaguen M, Labussière E (2017). Effects of dietary supplementation with freshwater microalgae on growth performance, nutrient digestibility and gut health in weaned piglets. Animal..

[21] Martins CF, Pestana JM, Ribeiro DM, Madeira MS, Alfaia CM, Lopes PA, Coelho D, Lemos JP, Almeida AM, Prates JAM, Freire JPB. Effect of dietary inclusion of Spirulina on production performance, nutrient digestibility and meat quality traits in post-weaning piglets. J Anim Physiol Anim Nutr. 2020*;00:*1*–*13 (E pub ahead of print) (10.1111/jpn.13470). 10.1111/jpn.13470 33210778

[22] Ovando CA, Carvalho JC, Pereira GVM, Jacques VTS, Soccol CR (2018). Functional properties and health benefits of bioactive peptides derived from *Spirulina*: a review. Food Rev Int.

[23] Belay A (2002). The potential application of Spirulina (Arthrospira) as a nutritional and therapeutic supplement in health management. J Am Nutraceutical Assoc.

[24] Oliveira WDC, De Oliveira CA, Campos-Galvão MEM, De Castro VC, Do Nascimento AG (2013). Cianobactérias: Uma revisão sobre potencial nutricional e alguns aspectos biotecnológicos. BBR.

[25] Jackson PGG, Cockcroft PD. Laboratory reference values: biochemistry. In: Clinical Examination of Farm Animals. Appendix 3. Oxford: Blackwell Science; 2002. p. 303-305.

[26] De Caire GZ, De Cano MS, De Mule CZ, Steyerthal N, Piantanida M. Effect of Spirulina platensis on glucose, uric acid and cholesterol levels in the blood of rodents. Intern J Exp Botany. 1995;57:93–6.

[27] Cheong SH, Kim MY, Sok DE, Hwang SY, Kim JH, Kim HR, Lee JH, Kim YB, Kim MR. Spirulina prevents atherosclerosis by reducing hypercholesterolemia in rabbits fed a high-cholesterol diet. J Nutr Sci Vitaminol. 2010;56:34–40. 10.3177/jnsv.56.34 20354344

[28] Rodrigues PO, Martins SV, Lopes PA, Ramos C, Miguéis S, Alfaia CM, Pinto RMA, Rolo EA, Bispo P, Batista I, Bandarra NM, Prates JAM. Influence of feeding graded levels of canned sardines on the inflammatory markers and tissue fatty acid composition of Wistar rats. Br J Nutr. 2014;112(3):309–19. 10.1017/S0007114514000853. 10.1017/S0007114514000853 24775714

[29] Spruijt J, van der Weide R, van Krimpen M. Opportunities for micro algae as ingredient in animal diets. Report from Project: Nutritional value of micro algae in diets for livestock. Application Centre for Renewable Resources. 2016.

[30] Karkos PD, Leong SC, Karkos CD, Sivaji N, Assimakopoulos DA (2011). *Spirulina* in clinical practice: evidence-based human applications. Evid-Based Compl Alt Med.

[31] Romay C, Armesto J, Remirez D, González R, Ledon N, Garcia I (1998). Antioxidant and anti-inflammatory properties of C-phycocyanin from blue-green algae. Inflamm Res.

[32] McMichael MA (2007). Oxidative stress, antioxidants, and assessment of oxidative stress in dogs and cats. J Am Vet Med A.

[33] Farag MR, Alagawany M, Abd El-Hack ME, Dhama K (2016). Nutritional and **healthical aspects** of Spirulina (Arthrospira) for poultry, animals and human. Int J Pharmacol.

[34] Waggiallah H, Alzohairy M (2011). The effect of oxidative stress on human red cells glutathione peroxidase, glutathione reductase level, and prevalence of anemia among diabetics. N Am J Med Sci.

[35] Behne D, Wolters W (1983). Distribution of selenium and glutathione peroxidase in the rat. J Nutr.

[36] Burke NC, Scaglia G, Boland HT, Swecker WS (2009). Influence of two-stage weaning with subsequent transport on body weight, plasma lipid peroxidation, plasma selenium, and on leukocyte glutathione peroxidase and glutathione reductase activity in beef calves. Vet Immunol Immunopathol.

[37] Nieto N, Lopez-Pedrosa JM, Mesa MD, Torres ML, Fernandez ML, Rios A, Suarez MD, Gil A (2000). Chronic diarrhea impairs intestinal antioxidant defense system in rats at weaning. Dig Dis Sci.

[38] Matés JM, Pérez-Gómez C, Núnez de Castro L (1999). Antioxidant enzymes and human diseases. Clin Biochem.

[39] Du L, He F, Kuang L, Tang W, Li Y, Chen D (2017). eNOS/iNOS and endoplasmic reticulum stress-induced apoptosis in the placentas of patients with preeclampsia. J Hum Hypertens.

[40] Amador P, Garcia-Herrera J, Marca MC, de la Osada J, Acin S, Navarro MA, Salvador MT, Lostao MP, Rodriguez-Yoldi MJ (2007). Intestinal D-galactose transport in an endotoxemia model in the rabbit. J Membr Biol.

[41] Nafikov RA, Beitz DC (2007). Carbohydrate and lipid metabolism in farm animals. J Nutr.

[42] Martins SV, Pires VMR, Madeira AP, Nascimento M, Alfaia CM, Castro MF, et al. Novel anti-adipogenic properties of the individual *trans*8,*cis*10 conjugated linoleic acid (CLA) isomer in 3T3-L1 adipocytes. Eur J Lipid Sci Technol. 2017;119(2). 10.1002/ejlt.201600042.

[43] Nakamura MT, Nara TY (2004). Structure, function, and dietary regulation of delta6, delta5, and delta9 desaturases. Annu Rev Nutr.

[44] Guzman C, Benet M, Pisonero-Vaquero S, Moya M, García-Mediavilla V, Martínez-Chantar ML, González-Gallego J, Castell JV, Sánchez-Campos S, Jover R (2013). The human liver fatty acid binding protein (FABP1) gene is activated by FOXA1 and PPARα; and repressed by C7EBPα: implications in FABP1 down-regulation in non-alcoholic fatty liver disease. Biochim Biophys Acta Mol Cell Biol Lipids.

[45] Van der Leij FR, Huijkman NC, Boomsma C, Kuipers JR, Bartelds B (2000). Genomics of the human carnitine acyltransferase genes. Mol Genet Metab.

[46] Poulsen LC, Siersbaek M, Mandrup S (2012). PPARs: fatty acid sensors controlling metabolism. Semin Cell Dev Biol.

[47] Correia CS, Alfaia CM, Madeira MS, Lopes PA, Matos TJS, Cunha LF, Prates JAM, Freire JPB (2017). Dietary inclusion of tomato pomace improves meta oxidative stability of young pigs. J Anim Physiol Anim Nutr.

[48] AOAC. 2000. Official methods of analysis. Assoc. Offic. Anal. Chem. 17th ed. Arlington, VA, USA. AOAC. Official methods of analysis. 17th ed. Arlington: Assoc. Offic Anal Chem; 2000.

[49] Sukhija PS, Palmquist DL (1988). Rapid method for determination of total fatty acid content and composition of feedstuffs and feces. J Agric Food Chem.

[50] Teimouri M, Amirkolaie AK, Yeganeh S (2013). The effects of *Spirulina platensis* meal as feed supplement on growth performance and pigmentation of rainbow trout (*Oncorhynchus mykiss*). Aquaculture.

[51] Hynstova V, Sterbova D, Klejdus B, Hedbavny J, Huska D, Adam V (2018). Separation, identification and quantification of carotenoids and chlorophylls in dietary supplements containing *Chlorella vulgaris* and *Spirulina platensis* using high performance thin layer chromatography. J Pharm Biomed Anal.

[52] Prates JAM, Quaresma MAG, Bessa RJB, Fontes CMGA, Alfaia CM (2006). Simultaneous HPLC quantification of total cholesterol, tocopherols and β-carotene in Barrosã-PDO veal. Food Chem.

[53] Friedewald WT, Levy RI, Fredrickson DS (1972). Estimation of the concentration of low-density lipoprotein cholesterol in plasma, without use of the preparative ultracentrifuge. Clin Chem.

[54] Covaci A, Voorspoels S, Thomsen C, van Bavel B, Neels H (2006). Evaluation of total lipids using enzymatic methods for the normalization of persistent organic pollutant levels in serum. Sci Total Environ.

[55] Folch J, Lees M, Stanley GH (1957). A simple method for the isolation and purification of total lipids from animal tissues. J Biol Chem.

[56] Carlson LA (1985). Extraction of lipids from human whole serum and lipoproteins and from rat liver tissue with methylene chloride-methanol: a comparison with extraction chloroform-methanol. Clin Chim Acta.

[57] Raes K, De Smet SD, Demeyer D (2001). Effect of double-muscling in Belgian blue young bulls on the intramuscular fatty acid composition with emphasis on conjugated linoleic acid and polyunsaturated fatty acids. Anim Sci.

[58] Alves SP, Bessa RJB (2009). Comparison of two gas–liquid chromatograph columns for the analysis of fatty acids in ruminant meat. J Chromatogr.

[59] Madeira MS, Pires VMR, Alfaia CM, Costa ASH, Luxton R, Doran O, Bessa RJB, Prates JAM (2013). Differential effects of reduced protein diets on fatty acid composition and gene expression in muscle and subcutaneous adipose tissue of Alentejana purebred and large white × landrace × Pietrain crossbred pigs. Br J Nutr.

[60] Livak KJ, Schmittgen TD (2001). Analysis of relative gene expression data using real-time quantitative PCR and the 2(−Delta C(T)) method. Methods..

[61] Fleige S, Walf V, Huch S (2006). Comparison of relative mRNA quantification models and the impact of RNA integrity in quantitative real-time RT-PCR. Biotechnol Lett.

[62] SAS Institute Inc. SAS/STAT 9.2 User’s Guide, 2nd ed. Cary, NC: SAS Institute Inc. 2009.63. Noblet J, Fortune H, Dubois S, Henry Y. Nouvelles bases d'estimation des teneurs en énergie digestible métabolisable et nette des aliments pour le porc. INRA, Station de Recherches Porcines Saint-Gilles 35590 L'Hermitage. 1989.

